# Public-private configuration in health systems and inequalities in Latin America: a scoping review

**DOI:** 10.1590/0102-311XEN260825

**Published:** 2026-08-10

**Authors:** Eduarda Ferreira dos Anjos, Adelyne Maria Mendes Pereira, Suelen Carlos de Oliveira, Maria del Pilar Flores-Quispe, Elzo Pereira Pinto, Cristiani Vieira Machado

**Affiliations:** 1 Instituto Gonçalo Muniz, Fundação Oswaldo Cruz, Fiocruz, Salvador, Brasil.; 2 Escola Nacional de Saúde Pública Sergio Arouca, Fundação Oswaldo Cruz, Fiocruz, Rio de Janeiro, Brasil.

**Keywords:** Health System Financing, Health Services Coverage, Health Equity, Public-Private Pathership, Latin America, Financiamento dos Sistemas de Saúde, Cobertura de Serviços de Saúde, Equidade em Saúde, Parcerias Público-Privadas, América Latina, Financiación de los Sistemas de Salud, Cobertura de los Servicios de Salud, Equidad en Salud, Asociación entre el Sector Público-Privado, América Latina

## Abstract

This study identifies and explores literature evidence on the effects of public-private partnerships in health systems on health inequities in Latin America. For this purpose, a scoping review was conducted following the Joanna Briggs Institute and PRISMA-ScR guidelines, employing the methodological steps of: (1) identifying the research question; (2) identifying relevant studies; (3) study selection; (4) data charting; and (5) collating, summarizing and reporting the results. Study analysis focused on the health systems of Argentina, Brazil, Chile, Colombia, Cuba, Ecuador, Mexico, Peru, and Uruguay, emphasizing financing, coverage, supply, and access. Initial bibliographic search identified 2,596 studies published on the topic between 2002 and 2024, of which 102 were included in this review. All analyzed studies reveal that the nature of public-private relations within Latin American health systems reinforces structural inequalities expressed in various dimensions which require context-appropriate policies, including sufficient financing, strengthening private sector regulation, and redistribution of resources and services that follow the geographical logic of social and health needs. In this context, overcoming the segmentation of coverage and access in favor of universalizing services and consolidating the right to health is essential.

## Resumo

Este estudo buscou identificar e explorar evidências na literatura sobre os efeitos das parcerias público-privadas em sistemas de saúde nas desigualdades em saúde na América Latina. Para isso, foi realizada uma revisão de escopo de acordo com as diretrizes do Instituto Joanna Briggs e do PRISMA-Sc, seguindo-se os seguintes passos metodológicos: (1) identificação da questão de pesquisa, (2) indexação dos estudos relevantes, (3) seleção de estudos, (4) mapeamento dos dados e (5) compilação, síntese e relato dos resultados. Esta análise focou nos sistemas de saúde na América Latina (Argentina, Brasil, Chile, Colômbia, Cuba, Equador, México, Peru e Uruguai), enfatizando-se financiamento, cobertura, oferta e acesso. A busca inicial identificou 2.596 estudos sobre o tema entre 2002 e 2024, dos quais 102 foram incluídos nesta revisão. Eles mostram que a natureza das relações público-privadas nos sistemas de saúde latino-americanos reforça desigualdades estruturais em várias dimensões, exigindo políticas adequadas a cada contexto (como financiamento suficiente, fortalecimento da regulamentação do setor privado e redistribuição de recursos e serviços) que sigam a lógica geográfica das necessidades sociais e de saúde. Assim, é essencial superar a segmentação da cobertura e do acesso em favor da universalização dos serviços e da consolidação do direito à saúde.

## Resumen

Este estudio tuvo como objetivo identificar y explorar la evidencia en la literatura sobre los efectos de las asociaciones público-privadas en los sistemas de salud respecto a las desigualdades en salud en América Latina. Para ello, se realizó una revisión de alcance según las directrices del Instituto Joanna Briggs y del PRISMA-Sc, siguiendo las siguientes etapas metodológicas: (1) identificar la pregunta de investigación, (2) indexar los estudios relevantes, (3) seleccionar los estudios, (4) mapear los datos y (5) recopilar, sintetizar y reportar los resultados. Este análisis se centró en los sistemas de salud de América Latina (Argentina, Brasil, Chile, Colombia, Cuba, Ecuador, México, Perú y Uruguay), con énfasis en la financiación, la cobertura, la oferta y el acceso. La búsqueda inicial identificó 2.596 estudios sobre el tema publicados entre 2002 y 2024, de los cuales 102 se incluyeron en esta revisión. Estos estudios demuestran que la naturaleza de las relaciones público-privadas en los sistemas de salud latinoamericanos refuerza las desigualdades estructurales en diversas dimensiones, lo que exige políticas adecuadas para cada contexto (como una financiación suficiente, el fortalecimiento de la regulación del sector privado y la redistribución de recursos y servicios) que sigan la lógica geográfica de las necesidades sociales y de salud. Por lo tanto, es fundamental superar la segmentación de la cobertura y el acceso en favor de universalizar los servicios y consolidar el derecho a la salud.

## Introduction

Economic constraints, democratic instability, and social inequalities have historically influenced the development of national health systems in Latin America ^1^. In many countries, healthcare was linked to the labor market but widespread informal employment meant that many relied on limited public health programs. Private sector expansion in economically active regions increased disparities in healthcare access which varied based on labor market participation and socioeconomic factors like class, income, race, gender, and place of residence ^2^.

Access segmentation, institutional fragmentation, and public-private relations in health financing, provision, and coverage influence health inequalities ^3^. Additionally, Latin American countries have different political, institutional, and territorial structures. Argentina, Brazil, and Mexico are federations; whereas Chile, Colombia, Peru, and Ecuador are unitary states, with different decentralization levels, coordination mechanisms, and capacity to implement health policies ^4^.

Argentina has a segmented, fragmented, and decentralized health system and faces challenges caused by regional inequalities and coordination problems. Brazil presents a universal health system with insufficient public funding, territorial and social inequalities, and a dynamic State-subsidized private sector ^5^. Mexico possesses a highly fragmented and underfunded system with inequalities in access and quality ^6^. Chile’s healthcare system is dual, comprising a public and a private segment with distinct clienteles ^7^. The Colombian health system is based on structured pluralism, a model segmented between contributory and subsidized regimes that reinforces social inequalities ^8^. Segmentation and fragmentation are also evident in the health systems of Peru and Ecuador, which offer social insurance for workers, public health services, and a private sector with heterogeneous access ^5^ ^,^ ^9^. The COVID-19 pandemic has brought additional challenges to Latin American health systems, in contexts marked by stratified patterns of social protection and inequality ^10^.

Despite distinct configurations, most health systems in Latin America face a common contradiction: pressures to expand private health markets against a backdrop of relatively scarce resources and structurally unequal societies. Amid tensions between efforts to strengthen public health systems and pro-market reforms, various public-private arrangements have historically emerged across countries. The relative scarcity of analyses mapping the landscape of interactions between the public and private sectors in healthcare across Latin American countries, and their effects on inequalities, justified a scoping review with this regional and thematic focus.

This article presents the results of a scoping review identified evidence on the effects of public-private relations in health systems on health inequalities in Latin America.

## Methods

A scoping review was conducted focusing on public-private configurations in Latin American health systems between 2002 and 2024, a period that encompassed reforms in this area. This method enables mapping the literature on a broad subject, exploring key aspects and gaps. Latin America was selected due to its deep inequalities and segmented health systems with significant private sector involvement.

The scope review followed the Joanna Briggs Institute (JBI) and PRISMA-ScR (*Preferred Reporting Items for Systematic Reviews and Meta-Analyses Extension for Scoping Reviews*) guidelines, comprising the following methodological steps: (1) identifying the research question; (2) identifying relevant studies; (3) study selection; (4) data charting; and (5) collating, summarizing and reporting the results. The protocol, registered on the Open Science Framework platform (is available at https://osf.io/rkzx3/?view_only=4594a2c52f2d47128d805fdd9dd1359b), and has been published at Anjos et al. ^11^. 

Our research question used the PCC mnemonic, in which: “Population” was defined as “Health Systems”; the “Concepts” were “Health Systems”, “Public-private mix”, and “Health inequalities”; and the “Context” was “Latin America,” with searches targeting Argentina, Brazil, Chile, Colombia, Ecuador, Mexico, and Peru. These seven countries account for 77.4% of the Latin American population and express different public-private patterns in their health systems.

Studies focused on the public-private configuration in Latin American health systems, published in English, Portuguese, or Spanish from January 2002 to March 2024, were included. Sources searched included the following databases: Virtual Health Library (VHL), Embase, PubMed, SciELO, Web of Science, and Scopus. Search terms combined keywords related to the health system (financing, coverage, provision, access), territorial location (Latin America or specific country names), and attributes of equality or equity. Box 1 summarizes the search strategy, research question, and concepts adopted. The Supplementary Material (https://cadernos.ensp.fiocruz.br/ojs/index.php/csp/article/view/12111/21006) describes the search strategy used for each database.

**Box 1:** Research question, key concepts applied to scope revision and search strategies.

RESEARCH QUESTION	WHAT ARE THE EFFECTS OF THE PUBLIC-PRIVATE CONFIGURATION OF HEALTH SYSTEMS ON HEALTH INEQUALITIES IN LATIN AMERICA?
Key concepts applied to scope revision	Health systems “*A set of political, economic and institutional relations responsible for conducting the processes related to the health of a given population, which take the form of organizations, rules and services aimed at achieving results consistent with the concept of health that prevails in society*” ^109^ (p. 89) Public-private configuration In health systems, “*institutions organized to respond to health demands and needs* (...) *are designated as public or private*” ^110^ (p. 118). The processes that characterize the organization of health systems and in which public or private institutions can operate are mainly financing, coverage, provision, and access to health services Health inequalities “*Health inequalities are the systematic, avoidable and unfair differences in health outcomes that can be observed between populations, between social groups within the same population or as a gradient across a population ranked by social position*” ^111^ (p. 22)
Search strategy	1. “Health system” OR “Health systems” OR “Health care system” OR “Health care systems” OR “Health policy” OR “Health polic*” OR “Health reform*” 2. “Public private” OR Public-private OR “Public and private” OR “Private sector” OR Privatization OR “Private plans” OR “Private insurance” 3. Financ* OR Spending OR Expense* OR Expenditures* OR Costs OR Spending OR Payment* OR Coverage OR Deliver* Or Provider* OR Regulation OR Access OR Utilization OR Provision 4. Latin America OR Brasil* OR Brazil OR Equador OR Ecuador OR México OR Mexico OR Chile OR Colômbia OR Colombia OR Peru OR Per* OR Argentin* 5. “Health inequalities” OR “Health disparities” OR “Health assymetries” OR “Health inequities”

Source: prepared by the authors.

All mapped documents were stored on Zotero, and the double-blind selection process was performed on Rayyan. Duplicates were removed using both software programs. After eliminating duplicates, eligibility was assessed by double-blind selection based on titles and abstracts, with conflicts resolved by a third reviewer.

Eligible files were imported into Excel to facilitate complete reading, descriptive analysis, and categorical analysis. Articles were manually excluded when not possible to access the full document (6), when they fell outside the scope review (13), when they had not undergone peer review (5), and when duplicates not detected by the software were identified (4). After review by an expert panel, four additional articles focusing on the public-private configuration in the health systems of Brazil (2), Colombia (1), and Chile (1), which met the eligibility criteria, were included.

All included articles underwent a thorough reading, descriptive analysis, and categorical analysis. Descriptive analysis provided information on the countries studied, the year and language of publication. Categorical analysis identified the categories of financing, coverage, access, and service provision. Articles that did not fit into these categories were grouped into a single category (“others”). Results were organized into graphs and tables.

A meeting was held with a panel of experts and stakeholders (researchers, health officials, health managers, and non-governmental representatives of social participation in health) to present and discuss the results before drafting the final version of the article.

## Results

**Figure 1:**
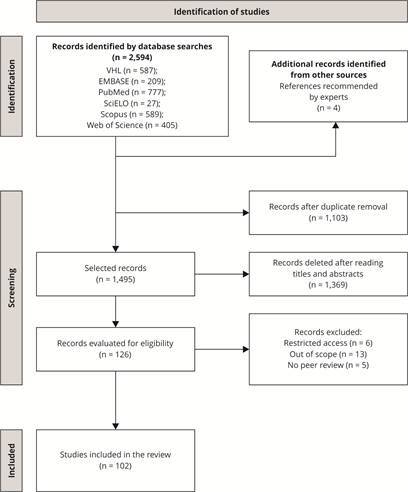
Steps for identifying, screening, and including studies according to the databases.

Source: prepared by the authors, with model adapted from Page et al. ^112^.

Of the 2,598 publications identified between 2002 and 2024, 2,594 were found by database searches and four recommended by experts, of which 1,103 were duplicates. After reviewing the titles and abstracts of 1,495 documents, 102 articles composed this review (Figure 1). Final selection of the 102 articles considered rigorous criteria of thematic relevance and specificity, prioritizing studies that presented empirical evidence or theoretical analyses focused on public-private interactions across the four structural dimensions of this study: financing, coverage, provision, and access. The Supplementary Material (https://cadernos.ensp.fiocruz.br/ojs/index.php/csp/article/view/12111/21006) provides a more detailed description of the sources included in this review.

**Figure 2:**
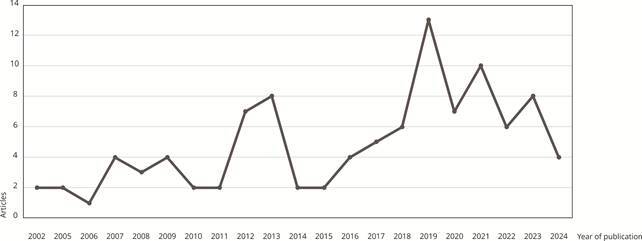
Distribution of articles by year of publication, 2002-2024.

Source: prepared by the authors based on the studies included in the review.

Distribution analysis of the 102 selected articles reveals fluctuations over time and a concentration of 80.1% publications between 2012 and 2024 (Figure 2). Most articles were published in English (64.7%), and cross-sectional (18.6%), cohort (10.8%), and ecological (6.9%) studies were prominent (data not shown).

Brazil (41.7%), Mexico (17.6%) and Colombia (17.6%) comprised the leading countries studied. Selected publications were categorized into four groups reflecting different aspects of the public-private configuration in health systems: financing, coverage, provision, and access to health services. Most studies focused on coverage (35.2%), financing (21.3%) and provision (20.4%). Studies that did not fit into the categories mentioned were included under “other” and analyzed separately. Table 1 presents this distribution by country and analysis category as a heat map.

**Table 1:** Distribution of articles by country and analytical category, 2002-2024 (heat map).

Country/Dimension	Financing	Coverage	Provision	Access	Others	Percentage
Latin America	3	1	1	0	1	5.6
Argentina	0	1	1	2	0	3.7
Brazil	11	18	9	4	3	41.7
Chile	1	1	3	0	0	4.6
Colombia	3	7	3	3	2	16.7
Cuba	0	0	2	0	0	1.9
Ecuador	0	1	0	2	1	3.7
Mexico	5	5	2	4	3	17.6
Peru	0	3	1	0	0	3.7
Uruguay	0	1	0	0	0	0.9
Total (percentage) *	21 (21.3)	38 (35.2)	22 (20.4)	15 (13.9)	10 (9.3)	100

Source: prepared by the authors based on the reviewed studies.

Note: the heat map shows the countries with the highest number of publications per year and categories, with darker shades of gray indicating higher intensity.

* The sum is greater than one hundred percent, since six articles addressed more than one category of analysis.

## Discussion

All analyzed studies emphasize the complexity of public-private configurations in Latin American health systems and the multifaceted, persistent health inequalities in the region driven by structural, political-institutional, and financial factors. Box 2 summarizes the analysis of main points regarding the effects and challenges of public-private configurations in health systems on health inequalities in Latin America.

**Box 2:** Effects and challenges of public-private configurations of health systems on health inequalities in Latin America.

DIMENSION	EFFECTS AND CHALLENGES
Health funding	Cuts in public spending have hold back reductions in infant mortality and other health indicators. Insufficient public funding, state subsidies and expansion of the private sector hinder the reduction of health inequalities National financing rules influence territorial inequalities High out-of-pocket health expenditures − especially for medicines − are detrimental to low-income families Outpatient and hospital co-payments represented a barrier to accessing health services Coordination between economic and social policies is essential to reduce health inequalities and maximize the impact of health actions
Health coverage	Coexistence of advances in coverage and structural limitations − such as segmentation − in Latin American health systems Dependence on the private sector, fragmentation of public policies, and socioeconomic disparities limit progress toward universality and equity Need to strengthen state regulation, integrate public and private services to serve the public interest, and prioritize redistributive strategies that guarantee access, quality, and continuity of care Transforming the structural foundations of systems is necessary to reduce inequalities and achieve health as a universal right
Health services provision	Spatial inequalities in the provision of specialized services − such as tests and intensive care beds − lead to poorer health outcomes in poor, peripheral, and rural regions Inequalities in the provision of complex services between the public and private sectors exacerbate inequalities in access among population groups
Healthcare access	Expansion of public provision, including in primary health care, has been associated with a reduction in inequalities in access to health services In countries with segmented systems, inequalities in access persist between populations covered or not covered by social security Greater difficulties in accessing health services for Black and Indigenous populations, who have worse health outcomes Inequalities in the provision of public and private services increase access barriers for vulnerable groups Inequalities in access are multifaceted, reflecting interactions between income, location, age group, and type of coverage Marked disparities in the health insurance and health plan segment reinforce the need for greater state regulation

Source: prepared by the authors, based on the articles included in the scoping review.

Regarding financing, the studies reported insufficient government spending on health ^12^, highlighting regional inequalities in financing and in spending between the public and private sectors ^13^ ^,^ ^14^. Latin American countries that restricted government spending on health were less likely to achieve infant mortality reduction goals ^15^. We found no evidence that state incentives for public-private partnerships reduced inequalities. On the contrary, their adoption in Latin American countries may increase them, as they prioritize the wealthier population and risk neglecting vulnerable groups ^16^.

In Brazil, increased public funding for primary health care (PHC) via per capita incentives and the Family Health Strategy (FHS) has expanded access to services, reduced hospitalizations for PHC-sensitive conditions, and lowered infant mortality across all regions of the country ^17^. However, austerity measures have introduced risks of setbacks in health outcomes and increased inequities ^18^ ^,^ ^19^. Focus on the state’s role in the Brazilian health system has brought progress to public funding ^20^, albeit insufficient, and private spending remains high ^21^ ^,^ ^22^ ^,^ ^23^. Colombia presents striking financing inequalities due to the segmented healthcare model ^24^. Mexico, compared with other middle-income countries, showed higher out-of-pocket health expenditures ^25^ and disparities in per capita spending across states ^26^. Chile lacked sufficient funding for its public system ^27^.

Medicine costs featured among the largest components of out-of-pocket expenditures, with lower-income households in Brazil incurring proportionally higher costs than higher-income households ^28^. Private health insurance costs also contribute to catastrophic expenditures among poor populations ^29^ ^,^ ^30^. In Chile, beneficiaries of Fondo Nacional de Salud (FONASA), a social security program for low-income individuals without formal employment, experienced a higher rate of catastrophic health expenditures than beneficiaries of Social Security Health Institution (ISAPRE, acronym in Spanish), which insures formal workers who choose this private regime ^31^. Colombia presented regional disparities in catastrophic health expenditures, with higher rates among populations lacking social security, rural residents, and lower-income households ^32^.

In Mexico, analyses of the Popular Health Insurance, which was in effect from 2003 to 2018 and targeted the population without access to social security, found a reduction in out-of-pocket expenses among those enrolled ^25^ ^,^ ^33^ but observed no effects on drug expenditures ^25^ ^,^ ^34^. The country also showed regional disparities in diabetes spending, with USD 51 of every USD 100 spent coming from user payments ^35^.

Regarding population coverage, disparities in labor market participation and social inequalities are more pronounced in contexts of high labor informality, unemployment, or poverty. A comparative study of Brazil and Turkey found that expanding comprehensive PHC coverage via multidisciplinary teams improved access equity in Brazil. However, challenges remain in tertiary care and in retaining professionals in rural or peripheral areas, and in regional disparities in public and private coverage ^36^. Other studies highlighted coverage inequalities across municipalities and social groups in Chile ^37^ and Brazil ^38^ ^,^ ^39^, revealing contradictions related to state funding of private plans for civil servants ^40^ and subsidies for private insurance coverage ^16^ ^,^ ^17^.

Argentina presented weaknesses in the decentralization of financing and service delivery, with inequalities in access and inefficient operation of the Obras Sociales program due to their large number leading to high administrative costs ^41^. Observed differences in mortality between public and private hospitals pointed to more severe patients admitted to public hospitals and a higher risk of septic shock in that group ^42^.

Chile’s health system showed no statistically significant association between coverage type (public or private) and the reduction in unmet healthcare needs (defined as not having a medical consultation for a health issue in the past 30 days) ^43^. Public insurance received lower average monthly funding despite the older population covered by the FONASA presenting greater healthcare needs, lower income, higher out-of-pocket expenses, and a lower likelihood of accessing services than those covered by the ISAPRE ^31^. People with private insurance had higher survival rates ^44^.

In Colombia, despite expanded coverage and benefit equalization, inequalities persist across health insurance regimes ^45^ and by gender, income, and ethnicity ^46^. Maternal mortality ^47^, risk of death from malnutrition in children under five, and the risk of malaria were higher among those enrolled in the subsidized system, who have worse socioeconomic status ^48^. The statistically significant association found between ethnic-racial status and coverage scheme revealed that Indigenous and Black populations are more likely to be enrolled in the subsidized regime ^49^.

Effective coverage (the share of potential health system benefits provided) in Mexico ranged from 54% in Chiapas, a poor state, to 65.1% in the Federal District. Effective coverage for the poorest quintile was 52% compared with 61% for the wealthiest quintile ^50^. Another study found variable risk of death among children with acute lymphoblastic leukemia depending on the type of health system coverage ^51^. Health insurance coverage alone was not sufficient to reduce health inequalities ^52^. Peru presented regional differences in average per capita coverage of medical consultations between wealthier and poorer areas ^53^.

As for service provision, evidence indicated structural fragmentation and segmentation in health systems exacerbated by poorly regulated privatization. Regional comparisons evinced that having multiple providers − public, private, and philanthropic − simultaneously increased disparities in primary care, hospital care, and health outcomes ^54^ ^,^ ^55^ ^,^ ^56^.

Health system fragmentation emerged a significant factor in health inequalities ^57^, especially during the COVID-19 pandemic. The policies implemented during the early months of the health crisis in Mexico varied across public health subsystems and were marked by a fragmented and inconsistent response, creating gaps that weakened the effectiveness of actions ^58^.

Health system segmentation has influenced disparities in morbidity and hospitalization patterns ^59^ ^,^ ^60^ ^,^ ^61^ ^,^ ^62^ and in health care outcomes. In Brazil, women with breast cancer had lower survival rates when treated at public hospitals than at private facilities ^63^. Service provision varied greatly in the private sector, but was more equitable among Brazilian Unified National Health System (SUS, acronym in Portuguese) users ^64^ ^,^ ^65^. Although recent reforms in Colombia have increased access to and utilization of health services, segmentation and inequalities persist ^66^.

Incremental reforms in Chile have attempted to mitigate the negative effects of segmented and fragmented healthcare, but they have not been sufficient to foster structural changes in inequalities ^27^. In Argentina, the healthcare system structure has deepened inequalities. Public hospitals have reported higher sepsis mortality rates linked to socioeconomic factors ^42^, and disparities in access and health outcomes varied by coverage type (social security or private insurance) ^41^. Studies in Peru have highlighted the role of the public sector in reducing inequalities via income transfer programs and the expansion of public health insurance ^67^ ^,^ ^68^.

Articles highlighted the spatial inequality in the provision of complex health services ^69^ ^,^ ^70^. In Brazil, intensive care beds are primarily concentrated in major cities and among private providers, undermining the needs of peripheral populations and hindering healthcare regionalization. In Ecuador, socioeconomic factors and hospital births were associated with territorial inequalities in maternal mortality ^71^.

Finally, unequal access to basic health services such as vaccination and prenatal care accentuates health disparities in Latin America. Concentration of benefits from economic and technological development among privileged groups widens inequalities ^6^.

The analyzed studies revealed persistent inequalities in service utilization shaped by socioeconomic, racial, age, and territorial factors, and by the type of coverage. Public-private configuration of service provision and income disparities influence the equity of care in Brazil ^72^ ^,^ ^73^ ^,^ ^74^. In Mexico, income was a significant factor shaping inequalities in access to, distribution of, and utilization of health services ^75^.

Among older adults, disparities in access lead to lower service utilization among the most vulnerable ^76^ ^,^ ^77^. Similarly, maternal and child health services, including neonatal hearing screening ^78^ and postpartum follow-up ^79^ ^,^ ^80^, present significant socioeconomic and regional inequalities.

Reproductive health care reveals significant racial inequalities, with Black women in Brazil receiving less qualified care than White women ^81^. In Colombia, the prevalence of opportunistic infections among insured people living with HIV was linked to socioeconomic factors ^82^. In Mexico and Colombia, the type of health insurance was associated with socioeconomic factors such as income and place of residence, and influenced clinical outcomes and survival rates for cancer patients. In both countries, individuals with metastatic renal and breast cancers who had less coverage or faced social vulnerability encountered treatment difficulties and had lower survival rates ^83^ ^,^ ^84^.

In Mexico, individuals without social security faced challenges in accessing ^85^ and using ^86^ health services, with income level linked to disparities in service utilization ^79^. Reforms implemented by the country favored urban over rural areas, and the allocation of resources improved system efficiency but did not guarantee a fair distribution across different regions and social groups ^87^.

In Argentina, the highest prevalence of hypoglycemia among people with diabetes treated in the public sector was associated with unemployment, low income, and limited health education, reinforcing the impact of social conditions and type of coverage on clinical outcomes ^73^.

In Peru, Comprehensive Health Insurance (SSI, acronym in Spanish) reduced inequalities in consultations ^68^, while structural reforms in Ecuador narrowed gaps between population groups ^88^. In Brazil, the Family Health Strategy (FHS) significantly reduced mortality risk among low-income urban populations, with more pronounced effects on the most racially and socioeconomically vulnerable groups ^89^.

Regarding access to health services for women in a Brazilian capital city, 24.7% of women aged 40 to 69 had never had a mammogram, a finding linked to social and racial inequalities ^81^. FHS coverage was associated with increased access to mammography in areas with lower private insurance coverage ^90^, underscoring the relevance of SUS. However, access inequalities based on race ^91^ ^,^ ^92^, social status, economic status ^93^, and between older adults with and without health insurance ^94^ persist in the country.

In the Colombian capital, women with breast cancer presented survival rates that varied by socioeconomic status and type of health insurance, with worse outcomes among the most vulnerable. This evinces that social factors influence equity in cancer care regardless of disease stage ^84^. In Ecuador, health reforms helped reduce gender gaps in women’s access to health services but socioeconomic disparities remained ^95^.

Access barriers also affected children, older adults, and other vulnerable groups. From 2003 to 2009, infant mortality rates in Colombia varied by department, with disparities associated with health insurance coverage and access to private services. This suggests that resource distribution is more affected by a region’s development level than by the population’s needs ^96^. Despite higher coverage, differences in usage of outpatient and preventive services among older adults persisted, influenced by income, insurance type, and residence location ^97^.

In Mexico, who aimed to implement universal coverage through the Popular Health Insurance program, demand for physical exams among adults rose. However, the program’s effects were limited or uneven among vulnerable groups such as women, children, and indigenous people, particularly in screening for diabetes and cervical cancer ^98^.

Some articles fit none of the specific analytical category. Although the COVID-19 pandemic has challenged health systems worldwide, few studies have examined public-private partnerships during the health crisis. In Brazil, COVID-19 mortality rates were associated with health inequalities ^99^, and private health plan beneficiaries in higher-income groups were more likely to use specialized services ^100^.

All analyzed studies also highlight the importance of health policy contexts. Despite reforms aimed at achieving universal health coverage, the Colombian population still present a poor perception of their health status and barriers to accessing health care persist ^101^. Research conducted in Mexico emphasized the need to account for epidemiological changes by increasing per capita spending ^102^ and addressing regional inequalities ^103^.

In Ecuador, neoliberal reforms have led to the adoption of biomedical approaches that overlook the population’s specific contexts and needs ^104^. In Brazil, the implementation of health technologies did not always align with health or territorial priorities and was shaped by the power and interests of private providers ^105^. Finally, public and private providers in Argentina differed in how they understood the population using their services, highlighting distinct challenges in accessing PHC across sectors ^106^.

Distinct health care system configurations across Latin American countries lead to disparities in health rights, quality, and access to services. Complex public-private arrangements favor the most profitable sectors, yet they vary across nations. In countries like Colombia and Mexico, formally segmented health systems add another layer of inequality between social groups. Meanwhile, addressing inequalities in Brazil requires strengthening the SUS and reducing state subsidies to the private sector, including health insurance companies and for-profit health care providers. Recent reforms adopted in Mexico deserve highlight, including the launch of IMSS-Bienestar in 2023 and the April 2026 announcement of a Universal Health Service that integrates health care access across the country’s social security institutions as first steps toward a unified and comprehensive system ^107^.

Despite its methodological rigor, our study has limitations. Scoping reviews may not identify all relevant articles due to the search strategies used. For example, our review retrieved no articles on financialization and health inequalities, or on specific types of private services such as pharmacy-linked practices in Mexico and autonomous private clinical practices in other countries. Additionally, the study focused on health inequalities without conceptually distinguishing between health inequities and health disparities, which may have limited our interpretation of the findings. Other limitations include an imbalance in scientific publications across the analyzed dimensions and variability in the scope of the reviewed articles. Our findings might not be generalizable to all Latin American nations due to regional diversity, the limited number of cross-country comparative studies, and imbalances in scientific production. Although Brazil ranks fifth in Latin America in terms of number of publications per 100,000 inhabitants (28.927), it leads in the total number of articles published and citations in the health policy field, with an H-index of 103, followed by Mexico (69), Argentina (66), Chile (54), and Colombia (52) ^108^, which explains the larger share of selected articles from this country.

## Conclusions

Our results indicate that public-private arrangements in Latin American health systems over recent decades have been shaped by structural factors, institutional legacies, and health reform processes. 

Brazil possesses a public, universal health system alongside a dynamic State-supported private sector, which represents a significant contradiction. In the other studied countries, health systems usually rely on employment status, specific public health programs, and various private sectors with a trend toward expansion. In most Latin American nations, health coverage is tied to economic and employment status, favoring higher-income groups who can access both public and private services. Socioeconomic inequalities, limited government regulation, subsidies, and dependence on the private sector hinder achieving universal and equitable healthcare.

Public funding is both insufficient to meet Latin American health needs and unevenly distributed across the region and among social groups. Fiscal austerity measures undermine social spending and can worsen health indicators. Health inequalities are exacerbated in many countries by high out-of-pocket expenses for medicines, co-payments that act as barriers to access, and government subsidies to the private sector.

Universal health coverage assumes that health systems should integrate public and private services to expand coverage; however, expanding formal coverage does not necessarily reduce inequalities in access, which depend on the public-private configuration of health systems. We found evidence that even when some health reforms expanded coverage − as in Colombia and Mexico −, shifts in state-market relations in health financing, regulation, and service delivery tend to deepen health disparities in the provision, access, and utilization of health services.

Our findings evince that in contexts driven by market interests and marked by power imbalances and inequalities across multiple dimensions − territorial, socioeconomic, and ethnic-racial −, policies to expand health coverage are seldom neutral. Some strategies can reinforce social stratification and health inequalities depending on their public-private arrangements. Latin American countries must invest in public health systems, strengthen government regulation of the private sector, subordinate it to the public good, and prioritize redistributive policies that enhance equity in funding, access, quality, and continuity of care. Linking economic and social policies and tailoring health policies to national contexts are essential for improving population health. Transforming Latin American health systems is crucial to reducing social inequalities and upholding health as a universal right.

## Data Availability

The sources of information used in the study are indicated in the body of the article.
