# Supplementary material for: Public-private configuration in health systems and inequalities in Latin America: a scoping review
Source: Cad Saude Publica. 2026 Aug 10;42(Suppl 1):e00260825. doi: 10.1590/0102-311XEN260825 (PMC13460911; doi:10.1590/0102-311XEN260825)
Supplement: Supplementary file 1 [file 1678-4464-csp-42-s1-EN260825-s.pdf]

Supplementary Material

Box S1 Search strategy.

Date of collection: April 17, 2024

**Search strategy axes**	
- Health systems<br>- Public/Private<br>- Financing<br>- Health inequalities<br>- Countries	
**General concept for constructing the search strategy**	
(Health systems) AND ((Public/Private) OR (Financing)) AND (Health inequalities) AND (Countries)	
**BVS**	**587 records**
**Strategy:** ("Health system" OR "Health systems" OR "Health care system" OR "Health care systems" OR "Health policy" OR "Health polic*" OR "Health reform*" OR "Unified Health System" OR sus OR "National Health Systems") AND (("Public private" OR public-private OR "Public and private" OR "Private sector" OR privatization OR "Private plans" OR "Private insurance" OR "Public Sector" OR "Public-Private Sector Partnerships" OR "Public-Private Mix") OR (financ* OR spending OR expense* OR expenditures* OR costs OR spending OR payment* OR coverage OR deliver* OR provider* OR regulation OR access OR utilization OR provision OR "Health Consortia" OR "Healthcare Financing" OR "Health Insurance")) AND ("Latin America" OR "Latinoamérica" OR "América Latina" OR brasil* OR brazil OR equador OR ecuador OR méxico OR mexico OR chile OR colômbia OR colombia OR peru OR perú OR peru* OR argentin*) AND ("Health inequalit*" OR "Health inequit*" OR "Health disparit*" OR "Health assymetr*" OR "Health Status Disparities" OR "Socioeconomic Disparities in Health" OR "Social Determinants of Health") AND (year_cluster:[2000 TO 2022])	
**Filters applied:**<br>▪ Title;<br>▪ Subject;<br>▪ Abstract;<br>▪ Period: 2000-2024	
**Embase**	**209 records**
**Strategy:** ('health system':ti,ab,kw OR 'health systems':ti,ab,kw OR 'health care system':ti,ab,kw OR 'health care systems':ti,ab,kw OR 'health policy':ti,ab,kw OR 'health polic*':ti,ab,kw OR 'health reform*':ti,ab,kw OR 'unified health system':ti,ab,kw OR sus:ti,ab,kw OR 'national health systems':ti,ab,kw) AND (('public private':ti,ab,kw OR public:ti,ab,kw) AND private:ti,ab,kw OR 'private sector':ti,ab,kw OR privatization:ti,ab,kw OR 'private plans':ti,ab,kw OR 'private insurance':ti,ab,kw OR 'public sector':ti,ab,kw OR 'public-private sector partnerships':ti,ab,kw OR 'public-private mix':ti,ab,kw OR financ*:ti,ab,kw OR expense*:ti,ab,kw OR expenditures*:ti,ab,kw OR costs:ti,ab,kw OR spending:ti,ab,kw OR payment*:ti,ab,kw OR coverage:ti,ab,kw OR deliver*:ti,ab,kw OR provider*:ti,ab,kw OR regulation:ti,ab,kw OR access:ti,ab,kw OR utilization:ti,ab,kw OR provision:ti,ab,kw OR 'health consortia':ti,ab,kw OR 'healthcare financing':ti,ab,kw OR 'health insurance':ti,ab,kw) AND ('latin america':ti,ab,kw OR 'latinoamérica':ti,ab,kw OR 'américa latina':ti,ab,kw OR brasil*:ti,ab,kw OR brazil:ti,ab,kw OR	

equador:ti,ab,kw OR ecuador:ti,ab,kw OR méxico:ti,ab,kw OR mexico:ti,ab,kw OR chile:ti,ab,kw OR colômbia:ti,ab,kw OR colombia:ti,ab,kw OR peru:ti,ab,kw OR perú:ti,ab,kw OR peru*:ti,ab,kw OR argentin*:ti,ab,kw) AND ('health inequalit*':ti,ab,kw OR 'health inequit*':ti,ab,kw OR 'health disparit*':ti,ab,kw OR 'health assymetr*':ti,ab,kw OR 'health status disparities':ti,ab,kw OR 'socioeconomic disparities in health':ti,ab,kw OR 'social determinants of health':ti,ab,kw) AND [2000-2022]/py

Filters applied:

- Title;
- Subject;
- Abstract;
- Period: 2000-2024

**PubMed**	**777 records**

Strategy: ("Health system" OR "Health systems" OR "Health care system" OR "Health care systems" OR "Health policy" OR "Health polic*" OR "Health reform*" OR "Unified Health System" OR SUS OR "National Health Systems") AND (("Public private" OR Public-private OR "Public and private" OR "Private sector" OR Privatization OR "Private plans" OR "Private insurance" OR "Public Sector" OR "Public-Private Sector Partnerships" OR "Public-Private Mix") OR (Financ* OR Spending OR Expense* OR Expenditures* OR Costs OR Spending OR Payment* OR Coverage OR Deliver* OR Provider* OR Regulation OR Access OR Utilization OR Provision OR "Health Consortia" OR "Healthcare Financing" OR "Health Insurance")) AND ("Latin America" OR "Latinoamérica" OR "América Latina" OR Brasil* OR Brazil OR Equador OR Ecuador OR México OR Mexico OR Chile OR Colômbia OR Colombia OR Peru OR Perú OR Peru* OR Argentin*) AND ("Health inequalit*" OR "Health inequit*" OR "Health disparit*" OR "Health assymetr*" OR "Health Status Disparities" OR "Socioeconomic Disparities in Health" OR "Social Determinants of Health")

Filters applied:

- All Fields;
- Period: 2000-2024

**Scielo**	**27 records**

Strategy: ("Health system" OR "Health systems" OR "Health care system" OR "Health care systems" OR "Health policy" OR "Health polic*" OR "Health reform*" OR "Unified Health System" OR SUS OR "National Health Systems") AND (("Public private" OR Public-private OR "Public and private" OR "Private sector" OR Privatization OR "Private plans" OR "Private insurance" OR "Public Sector" OR "Public-Private Sector Partnerships" OR "Public-Private Mix") OR (Financ* OR Spending OR Expense* OR Expenditures* OR Costs OR Spending OR Payment* OR Coverage OR Deliver* OR Provider* OR Regulation OR Access OR Utilization OR Provision OR "Health Consortia" OR "Healthcare Financing" OR "Health Insurance")) AND ("Latin America" OR "Latinoamérica" OR "América Latina" OR Brasil* OR Brazil OR Equador OR Ecuador OR México OR Mexico OR Chile OR Colômbia OR Colombia OR Peru OR Perú OR Peru* OR Argentin*) AND ("Health inequalit*" OR "Health inequit*" OR "Health disparit*" OR "Health assymetr*" OR "Health Status Disparities" OR "Socioeconomic Disparities in Health" OR "Social Determinants of Health")

Filters applied:

- All Fields;
- Period: 2000-2024

**Scopus**	**589 records**

Strategy: ("Health system" OR "Health systems" OR "Health care system" OR "Health care systems" OR "Health policy" OR "Health polic*" OR "Health reform*" OR "Unified Health System" OR SUS OR "National Health Systems") AND (("Public private" OR Public-private OR "Public and private" OR "Private sector" OR Privatization OR "Private plans" OR "Private insurance" OR "Public Sector" OR "Public-Private Sector Partnerships" OR "Public-Private Mix") OR (Financ* OR Spending OR Expense* OR Expenditures* OR Costs OR Spending OR

Payment* OR Coverage OR Deliver* OR Provider* OR Regulation OR Access OR Utilization OR Provision OR "Health Consortia" OR "Healthcare Financing" OR "Health Insurance")) AND ("Latin America" OR "Latinoamérica" OR "América Latina" OR Brasil* OR Brazil OR Equador OR Ecuador OR México OR Mexico OR Chile OR Colômbia OR Colombia OR Peru OR Perú OR Peru* OR Argentin*) AND ("Health inequalit*" OR "Health inequit*" OR "Health disparit*" OR "Health assymetr*" OR "Health Status Disparities" OR "Socioeconomic Disparities in Health" OR "Social Determinants of Health")	
**Filters applied:**<br>▪ Title;<br>▪ Subject;<br>▪ Abstract;<br>▪ Period: 2000-2024	
**Web of Science**	**405 records**
**Strategy:** (ALL=(("Health system" OR "Health systems" OR "Health care system" OR "Health care systems" OR "Health policy" OR "Health polic*" OR "Health reform*" OR "Unified Health System" OR SUS OR "National Health Systems") AND (("Public private" OR Public-private OR "Public and private" OR "Private sector" OR Privatization OR "Private plans" OR "Private insurance" OR "Public Sector" OR "Public-Private Sector Partnerships" OR "Public-Private Mix") OR (Financ* OR Spending OR Expense* OR Expenditures* OR Costs OR Spending OR Payment* OR Coverage OR Deliver* OR Provider* OR Regulation OR Access OR Utilization OR Provision OR "Health Consortia" OR "Healthcare Financing" OR "Health Insurance")) AND ("Health inequalit*" OR "Health inequit*" OR "Health disparit*" OR "Health assymetr*" OR "Health Status Disparities" OR "Socioeconomic Disparities in Health" OR "Social Determinants of Health"))) AND ALL=(("Latin America" OR "Latinoamérica" OR "América Latina" OR Brasil* OR Brazil OR Equador OR Ecuador OR México OR Mexico OR Chile OR Colômbia OR Colombia OR Peru OR Perú OR Peru* OR Argentin*))	
**Filters applied:**<br>▪ All Fields;<br>▪ Period: 2000-2024	
	**Total 2594 records**

Source: prepared by the authors

Box S2 Studies included in the review, Latin America, 2002 to 2024.

YEAR OF PUBLICATION	AUTHOR	TITLE	OBJECTIVE	COUNTRIES STUDIED	DIMENSION OF ANALYSIS
2002	Laurell AC, Soares LT.	*Serviços de saúde em grandes cidades latinoamericanas: o caso da cidade do México/DF*	Este artigo apresenta a atual política de saúde e a experiência de gestão dos serviços de saúde da Cidade do México, por parte da Secretaria de Saúde do Governo do Distrito Federal	Mexico	Access
2002	Casas-Zamora JÁ.	*Salud, desarrollo humano y gobernabilidad en America Latina y el Caribe a inicios del siglo XXI*	Este artículo analiza los conceptos de desarrollo humano sostenible, los determinantes sociales de la salud en general y las desigualdades en materia de salud en particular (género, origen étnico, nivel de ingresos), así como la relación entre la salud y el crecimiento económico a mediano y largo plazo	Latin America and Caribbean	Access
2005	Bahia L.	*Padrões e mudanças no financiamento e regulação do Sistema de Saúde Brasileiro: impactos sobre as relações entre o público e privado*	Sistematiza as matrizes interpretativas envolvidas no debate sobre o sistema de saúde brasileiro e avança hipóteses sobre as extensas e dinâmicas fronteiras de interseção público-privadas que o caracterizam	Brazil	Financing
2005	Muñoz-Hernández O, Garduño-Espinosa J, Pérez-Cuevas R.	*Higia y Panacea en la salud y la sociedad. Los sistemas de salud, un producto de la evolución social*	Este artículo analiza la influencia de algunos de estos factores determinantes en la salud de la población cubierta por el Instituto Mexicano del Seguro Social (IMSS), tales como los cambios demográficos (disminución de la tasa de natalidad, envejecimiento y aumento de la migración temporal), los cambios económicos (incorporación progresiva de las mujeres al mercado laboral) y los aspectos relacionados con el estilo de vida	Mexico	Others
2006	Castro MSM.	*Desigualdades sociais no uso de internações hospitalares no Brasil: o que mudou entre 1998 e 2003*	O objetivo deste artigo foi comparar os fatores associados às internações hospitalares no Brazil em 1998 e 2003	Brazil	Coverage
2007	Instituto de Pesquisa Econômica Aplicada	*Boletim políticas sociais: acompanhamento e análise*	Nesta edição, analisa-se a política de saúde no Brazil nos últimos dez anos, retornando, em alguns pontos, ao começo da década de 1990	Brazil	Coverage
2007	Pessoto UC, et al.	*Desigualdades no acesso e utilização dos serviços de saúde na Região Metropolitana de São Paulo*	Este artigo relata achados dos estudos de casos realizados em cinco municípios da Região Metropolitana de São Paulo (RMSP): São Paulo, Guarulhos, Santo André, Osasco e Franco da Rocha	Brazil	Provision
2007	Lozano R, et	*Benchmarking of*	In this article, we describe the steps	Mexico	Provision/

	al.	*performance of Mexican states with effective coverage*	taken to implement this performance benchmarking approach and present the main results for 2005-2006. We discuss the implications for the evolution of the Mexican health care system and other health care systems		Others
2007	Ruiz F, Amaya L, Venegas S.	*Progressive segmented health insurance: Colombian health reform and access to health services*	A prospective study was conducted to monitor the use of health care services and out-of-pocket expenses, using a cohort design	Colombia	Provision
2008	Barros AJD, Bertoldi AD.	*Out-of-pocket health expenditure in a population covered by the Family Health Program in Brazil*	This study was designed to assess the incidence of high health care costs in a low-income population, as well as the pattern of out-of-pocket health care payments	Brazil	Financing
2008	Palma-Solís M, et al.	*Political and social context of not attaining the Millennium Development Goal to reduce poverty.*	This study aims to explore the influence of the political and social context on the eradication of poverty	Americas	Financing
2008	Louvison MCP, et al.	*Desigualdades no acesso e utilização de serviços de saúde por idosos em São Paulo, Brasil*	Descrever fatores associados às desigualdades no acesso e utilização de serviços de saúde por idosos	Brazil	Coverage
2009	Palma-Solís MA, et al.	*State downsizing as a determinant of infant mortality and achievement of Millennium Development Goal 4.*	The objective of this study was to assess the global impact of government workforce reduction policies on the achievement of the UN's Millennium Development Goal 4 (MDG 4) regarding infant mortality rates	Latin America	Financing
2009	Matijasevich A, et al.	*Inequities in maternal postnatal visits among public and private patients: 2004 Pelotas cohort study*	The objectives of this study were: 1) to investigate inequalities in postnatal maternal care visits, 2) to examine differences in postnatal care coverage between public and private providers, and 3) to explore the relationship between the absence of postnatal maternal care visits and exclusive breastfeeding, contraceptive use, and maternal smoking three months after birth	Brazil	Coverage
2009	Homedes N, Ugalde A.	*Twenty-five years of convoluted health reforms in Mexico.*	This study examines 25 years of health care reform in Mexico and argues that, although costs and accessibility have increased, health inequalities, efficiency, productivity, and the quality of care have not improved	Mexico	Financing
2009	Linares-Pérez N, López-Arellano O.	*Los efectos de las transformaciones del sistema público de salud sobre la equidad en Mexico, 1995-*	Identificar los posibles efectos sobre la equidad en salud que puedan estar relacionados con las «intervenciones de reforma» llevadas a cabo en México entre 1995 y 2005	Mexico	Access

		2002			
2010	Yazlle-Rocha JS, Martinez EZ, Guedes GLM.	*Estudos das desigualdades na assistência hospitalar pela análise de correspondências*	Estudar as diferenças da morbidade hospitalar entre grupos sociais, considerando a ocupação do paciente e a fonte de financiamento da internação	Brazil	Coverage
2010	Yavich N, Báscolo EP, Haggerty J.	*Construyendo un marco de evaluación de la atención primaria de la salud para Latinoamérica*	Determinar la claridad, la relevancia y la idoneidad de una estrategia de evaluación de la atención primaria de salud en diferentes subsistemas de salud	Latin America	Others
2011	Silva HP, Viana AL.	*Health technology diffusion in developing countries: a case study of CT scanners in Brazil.*	This article examines the institutional environment in which decisions are made regarding the adoption of expensive medical devices in the Brazilian healthcare system	Brazil	Others
2011	Boing AC, Bertoldi AD, Peres KG	*Desigualdades socioeconômicas nos gastos e comprometimento da renda com medicamentos no Sul do Brasil*	Descrever as desigualdades socioeconômicas quanto ao uso, aos gastos e à renda comprometida com a compra de medicamentos	Brazil	Financing
2012	Lages RB, et al.	*Desigualdades associadas à não realização de mamografia na zona urbana de Teresina-Piauí-Brasil, 2010-2011*	Analisar o percentual de mulheres que não realizaram mamografia segundo variáveis socioeconômicas e demográficas em mulheres de 40 a 69 anos em Teresina, Piauí, Brasil	Brazil	Access
2012	Esteves RJF.	*The quest for equity in Latin America: A comparative analysis of the health care reforms in Brazil and Colombia*	This study aims to clarify why they pursued different strategies and what that meant in terms of health outcomes	Brazil and Colombia	Financing
2012	Lima-Costa M, et al.	*Mudanças em dez anos das desigualdades sociais em saúde dos idosos brasileiros (1998-2008)*	Analisar mudanças nos diferenciais por renda nas condições de saúde e no uso de serviços de saúde por idosos brasileiros	Brazil	Coverage
2012	Hernandes E, et al.	*Idosos com e sem plano de saúde e características socioepidemiológicas associadas*	Analisar fatores epidemiológicos e sociodemográficos associados à saúde de idosos com ou sem plano de saúde	Brazil	Provision
2012	Linares-Pérez N, López-Arellano O.	*Inequalities in health in Mexico*	This study presents a critical analysis of health sector reform in Mexico and its impact on access and equity in state health systems	Mexico	Others
2012	Maria Del Pilar CP, Cameron BL, Smith DG.	*Neoliberal-oriented health care system answer to global competition or a*	The purpose of this article is to explore how a neoliberal-oriented healthcare system affects the experiences of people living with chronic illnesses	Colombia	Access

		threat to health equality for people with chronic illness			
2012	Bernal O, Forero JC, Forde I.	*Colombia's response to healthcare crisis.*	Describe the origins of the crisis and explore the extent to which the reforms that followed are likely to ensure better health care for the population	Colombia	Others
2013	Kierans C, et al.	*When health systems are barriers to health care: Challenges faced by uninsured Mexican kidney patients*	The objective of this study is to document the challenges faced by poor, uninsured Mexican families when trying to access Renal Replacement Therapy (RRT)	Mexico	Access
2013	Jaramillo-Mejía MC, Chernichovsky D, Jiménez-Moleón JJ.	*Brechas regionales de la mortalidad infantil en Colombia*	Estudiar las variaciones en la tasa de mortalidad infantil (TMI) en los 33 departamentos administrativos de Colombia durante el período de 2003 a 2009, analizar la persistencia de las variaciones entre los departamentos a lo largo del tiempo y relacionar dichas variaciones con el impacto de las condiciones socioeconómicas y la disponibilidad de atención médica en la TMI	Colombia	Access
2013	Almeida G, et al.	*Analysis of the evolution and determinants of income-related inequalities in the Brazilian health system, 1998 - 2008.*	To analyze the trends and determinants of income-related inequalities in the Brazilian health care system between 1998 and 2008	Brazil	Coverage
2013	Ruiz-Gómez F, Zapata-Jaramillo T, Garavito-Beltrán L.	*Colombian health care system: results on equity for five health dimensions, 2003 - 2008*	To assess changes in five dimensions of health equity in the Colombian health system: health status, health insurance coverage, health service utilization, quality, and health expenditures	Colombia	Others
2013	Barraza-Lloréns M, Panopoulou G, Díaz BY.	*Income-related inequalities and inequities in health and health care utilization in Mexico, 2000 - 2006*	To measure income-related inequalities and inequities in health outcomes and the use of health services in Mexico	Mexico	Coverage
2013	Petrera M, et al.	*Equity in health and health care in Peru, 2004 - 2008*	This study assesses whether recent positive economic trends and pro-poor health policies have led to greater health equity and explores key factors that explain this change	Peru	Coverage
2013	Arredondo A, Reyes G.	*Health Disparities from Economic Burden of Diabetes in Middle-income Countries: Evidence from Mexico*	To quantify health disparities resulting from the economic burden of diabetes in Mexico	Mexico	Financing
2013	Martínez-	*Inequalities in Pap*	To examine the risk factors	Brazil	Coverage

	Mesa J, et al.	*smear screening for cervical cancer in Brazil*	associated with never having undergone cervical cancer screening (CCS) in Brazil		
2014	Martínez Valle A, Terrazas P, Alvarez F.	*Cómo reducir las inequidades de salud actuando sobre sus determinantes sociales: el papel del sector salud en Mexico.*	Analizar las medidas adoptadas en México por el sector de la salud entre 2007 y 2012 para combatir las desigualdades en salud, centrándose en los determinantes sociales	Mexico	Financing
2014	Frenz P, et al.	*Achieving effective universal health coverage with equity: evidence from Chile*	We analyzed data from household surveys conducted before and after the AUGE reform to estimate changes in levels of unmet health care needs − defined as the absence of a medical consultation for a health problem that occurred in the past 30 days − by age, sex, income, education, health insurance, place of residence, and ethnicity; we adjusted logistic regression models and used predictive margins	Chile	Provision
2015	Cotlear D et al.	*Overcoming social segregation in health care in Latin America*	This article in the series describes four phases in the history of Latin American countries that explain the roots of segmentation in health care and outlines three paths taken by countries seeking to overcome it: unification of the funds used to finance social security and the services of the Ministry of Health (a single public payer); free choice of provider or insurer; and expansion of services for the poor and the non-wage-earning population, making explicit the health care benefits to which all citizens are entitled	Cuba and Latin America	Provision
2015	Serrate PCF, Abreu EÁG, Lefrán AL.	*Universal health coverage and conciliation of criteria on the basis of Cuban experience*	This article aimed to provide insights into the current state of universal health coverage and how it can be aligned with a common approach based on the Cuban experience	Cuba	Provision
2016	Amaya-Lara JL.	*Catastrophic expenditure due to out-of-pocket health payments and its determinants in Colombian households*	The objective of this study was to identify the incidence and determinants of catastrophic out-of-pocket payments for medical care in Colombia	Colombia	Financing
2016	Bedregal P, et al.	*Desigualdades en desarrollo infantil temprano entre prestadores públicos y privados de salud y factores asociados en la Región Metropolitana de Chile*	Comparar el desarrollo en la primera infancia y los factores asociados en niños en edad preescolar de los sectores de salud pública y privada al inicio del estudio	Chile	Coverage

2016	Berquó E, Lago TG.	*Atenção em saúde reprodutiva no Brasil: eventuais diferenciais étnico-raciais*	Este estudo teve por objetivo comparar indicadores da atenção à saúde reprodutiva das mulheres negras e brancas	Brazil	Coverage
2016	Manica ST, et al.	*Desigualdades socioeconômicas e regionais na cobertura de exames citopatológicos do colo do útero*	Identificar as desigualdades socioeconômicas e regionais na cobertura do exame Papanicolau no estado do Rio Grande do Sul	Brazil	Provision
2017	Lima LD, et al.	*Arranjos de governança da assistência especializada nas regiões de saúde do Brasil*	Analisar os arranjos de governança regional da assistência especializada no SUS	Brazil	Financing
2017	Castillo-Laborde C et. al.	*Health insurance scheme performance and effects on health and health inequalities in Chile*	To evaluate the performance of Chilean public and private health insurance plans and their effects on health inequalities	Chile	Provision
2017	Alshamsan R, et al.	*Comparative health system performance in six middle-income countries: cross-sectional analysis using World Health Organization study of global ageing and health*	To assess and compare the performance of health care systems in six middle-income countries that are strengthening their health care systems in pursuit of universal health coverage	Mexico and six other middle-income countries	Financing
2017	Houweling TA, et al.	*Health insurance coverage, neonatal mortality and caesarean section deliveries: an analysis of vital registration data in Colombia*	We examined the association between health insurance coverage and neonatal mortality in Colombia	Colombia	Coverage
2017	Cárdenas WIL, Pereira AMM, Machado CV	*Trajetória das relações público-privadas no sistema de saúde da Colômbia de 1991 a 2015*	Analisar os arranjos público-privados no sistema de saúde da Colômbia entre 1991 e 2015, abarcando as dimensões de asseguramento e financiamento	Colombia	Financing/ Coverage
2018	Rasella D, et al.	*Child morbidity and mortality associated with alternative policy responses to the economic crisis in Brazil: A nationwide microsimulation study.*	We investigated how a reduction in the coverage of the PBF and ESF − compared to an alternative scenario in which the level of social protection provided by these programs is maintained − might affect the under-five mortality rate (U5MR) and socioeconomic inequalities in child health in Brazil through 2030, the target year for the Sustainable Development Goals	Brazil	Financing
2018	Matos	*Saúde da*	Realizar um diagnóstico situacional	Brazil	Provision

	CCSA, et al.	*População Negra: como nascem, vivem e morrem os indivíduos pretos e pardos em Florianópolis (SC)*	das condições de saúde da população negra no município de Florianópolis (SC)		
2018	Ramos ACV, et al.	*Estratégia Saúde da Família, saúde suplementar e desigualdade no acesso à mamografia no Brasil*	Avaliar a associação entre o acesso à mamografia no Brazil e a cobertura pela Estratégia Saúde da Família (ESF) e pela saúde suplementar	Brazil	Provision
2018	Rubinstein A, et al.	*Making Universal Health Care Effective in Argentina: A Blueprint for Reform*	In this article, we present our proposals for promoting CUS in Argentina	Argentina	Provision
2018	Knox M.	*Creating a preference for prevention: the role of universal health care in the demand for preventive care among Mexico's vulnerable populations.*	This article examines the effect of the SP on those at higher risk of health disparities, looking at the program's impact on the demand for preventive care services, particularly among women, children, and indigenous people	Mexico	Access
2018	Santos P, et al.	*Diferenciais dos aglomerados de nascidos vivos no Município de São Paulo, Brasil, 2010*	O objetivo do estudo foi identificar aglomerados de nascimentos segundo o tipo de hospital (SUS e não SUS) e a existência de diferenciais quanto a características socioeconômicas, materno-infantis e de acesso, medidos pela distância entre as residências maternas e os hospitais onde se deram os partos	Brazil	Coverage
2019	Hilarión-Gaitán L, et al.	*Inequalities in health by regime of affiliation to the health system in events of obligatory notification, Colombia, 2015*	Our objective was to estimate social inequalities in health in Colombia, based on the type of health insurance coverage as a proxy for socioeconomic status	Colombia	Provision
2019	Estenssoro E, et al.	*Health inequities in the diagnosis and outcome of sepsis in Argentina: a prospective cohort study.*	Our objective was to analyze differences in mortality between public and private hospitals, using the Sepsis-3 definitions	Argentina	Coverage
2019	Granda M L, Jimenez WG.	*The evolution of socioeconomic health inequalities in Ecuador during a public health system reform (2006-2014).*	This study assesses the progress made in addressing socioeconomic health inequalities before and after the reforms, between 2006 and 2014	Ecuador	Access
2019	Alonso F, et al.	*Reducing Health Inequalities:*	We assessed whether the healthcare reform altered early and long-term	Chile	Provision

		Comparison of Survival After Acute Myocardial Infarction According to Health Provider in Chile.	survival rates for AMI by hospital provider (public/private) using a population-based longitudinal study of patients aged 15 years or older who had a first AMI in Chile between 2002 and 2011		
2019	Cunha C, et al.	*Planos privados de saúde e a saúde dos trabalhadores do Brasil*	O presente estudo objetivou comparar as mudanças nos padrões de cobertura por planos privados de saúde entre servidores públicos, militares e demais trabalhadores no Brazil e verificar se a vinculação a planos de saúde repercute nas respectivas prevalências autorreferidas de Hipertensão Arterial Sistêmica (HAS) e Diabetes Mellitus (DM) no país	Brazil	Provision
2019	Castro-Ríos A, et al.	*Socioeconomic inequalities in survival of children with acute lymphoblastic leukemia insured by social security in Mexico: a study of the 2007-2009 cohorts.*	Socioeconomic inequalities in the survival of children (< 15 years of age) enrolled in the IMSS were analyzed	Mexico	Provision
2019	Machado, Cristiani Vieira; Silva, Gulnar Azevedo E	*Political struggles for a universal health system in Brazil: successes and limits in the reduction of inequalities*	This study analyzes the political struggles surrounding the implementation of a universal health care system from the mid-1980s to the present, and their effects on selected health indicators, focusing on relevant international and national contexts, political agendas, government policies, and key actors	Brazil	Financing
2019	Lima LD, et al.	*Arranjos regionais de governança do Sistema Único de Saúde: diversidade de prestadores e desigualdade espacial na provisão de serviços*	O estudo analisa os arranjos regionais de governança do Sistema Único de Saúde (SUS), segundo esfera jurídica dos prestadores e distribuição espacial da produção de serviços de média e alta complexidade no Brasil	Brazil	Coverage
2019	Lopera M, Lemos Y.	*Prevalence of opportunistic infections in insured patients with HIV and their association with socioeconomic and clinical factors in Colombia, 2012*	To estimate the prevalence of opportunistic infections among insured HIV patients and to determine their association with sociodemographic and clinical factors	Colombia	Coverage
2019	Gonçalves C, et al.	*Frequência e fatores associados à não realização da consulta*	Identificar a frequência da não realização das consultas de puerpério e fatores associados em mulheres residentes de um	Brazil	Coverage

		puerperal em um estudo de coorte	município de médio porte no Brasil		
2019	Friederic K, Burke BJ.	*La Revolución Ciudadana and social medicine: Undermining community in the state provision of health care in Ecuador.*	En este artículo, demostramos que las reformas de salud centradas en el Estado han debilitado, paradójicamente, la organización comunitaria para la salud colectiva	Ecuador	Others
2019	Cazzo E, Ramos AC, Chaim EA.	*Bariatric Surgery Offer in Brazil: a Macroeconomic Analysis of the Health system's Inequalities.*	To analyze the proportion of bariatric surgeries performed through the public health system in Brazil and to assess the influence of macroeconomic variables over time	Brazil	Financing
2019	Gomes MB, et al.	*Relationship between health care insurance status, social determinants and prevalence of diabetes-related microvascular complications in patients with type 1 diabetes: a nationwide survey in Brazil.*	To assess the relationship between social determinants, health insurance status, and the occurrence of chronic diabetes-related complications (CDRC) in Brazilian patients with type 1 diabetes	Brazil	Coverage
2020	Caeiro G, et al.	*Type 2 diabetes: Prevalence of hypoglycemia in public versus private health care system*	This study compares the prevalence of hypoglycemia among patients with type 2 diabetes (T2D) in the public versus private sectors and its relationship to social vulnerability	Argentina	Access
2020	Bedoya-Pacheco SJ, et al.	*Desigualdades na terapia intensiva no Rio de Janeiro: efeitos da distribuição espacial dos serviços de saúde na infecção respiratória aguda grave*	Analisar a distribuição das unidades de terapia intensiva para adultos, segundo a região geográfica e o setor sanitário no Rio de Janeiro, e investigar a mortalidade por infecção respiratória aguda grave no setor público e sua associação com a capacidade de terapia intensiva no setor público	Brazil	Coverage
2020	Quizhpe E, et al.	*Socioeconomic inequalities in women's access to health care: Has Ecuadorian health reform been successful?*	This study assesses whether the health reforms implemented between 2007 and 2017 helped reduce socioeconomic inequalities in women's access to health care	Ecuador	Access
2020	Figueiredo AM, et al.	*Determinantes sociais da saúde e infecção por COVID-19 no Brasil: uma análise da epidemia*	Analisar a influência de fatores socioeconômicos, demográficos, epidemiológicos e da estrutura do sistema de saúde na evolução da pandemia de COVID-19 no Brasil	Brazil	Others
2020	Massuda A.	*Mudanças no financiamento da Atenção Primária*	Neste artigo, discute-se os possíveis efeitos da nova política a partir de uma breve análise de contexto sobre	Brazil	Financing

		à Saúde no Sistema de Saúde Brasileiro: avanço ou retrocesso?	tendências globais de financiamento de sistemas de saúde e de remuneração por serviços de saúde, bem como dos avanços, desafios e ameaças à APS e ao SUS		
2020	Hone T, et al.	*Primary healthcare expansion and mortality in Brazil's urban poor: A cohort analysis of 1.2 million adults.*	This study assesses the impact of an ambitious program to rapidly expand ESF coverage in the city of Rio de Janeiro, Brazil, since 2008	Brazil	Access
2020	Garcia-Ramirez J, Nikoloski Z, Mossialos E.	*Inequality in healthcare use among older people in Colombia*	In this study, we assessed disparities in the use of health care services among older adults in Colombia	Colombia	Access
2021	Viáfara-López CA, Palacios-Quejada G, Banguera-Obregón A.	*Ethnic-racial inequity in health insurance in Colombia: a cross-sectional study.*	To characterize the relationship between ethnic and racial inequality and type of health insurance in Colombia	Colombia	Provision
2021	Ruano AL, et al.	*Understanding inequities in health and health systems in Latin America and the Caribbean: a thematic series.*	In this first collection of articles, we show how the region has approached − with varying degrees of success − the implementation of innovative reforms in its health care systems with the aim of strengthening governance, participation, and the response to the growing epidemiological and demographic demands of its diverse population	Latin America	Coverage
2021	Costa-Font J, Cowell FA, Saenz-de-Miera B.	*Measuring pure health inequality and mobility during a health insurance expansion: Evidence from Mexico.*	This article draws on new measures of pure (univariate) inequality and mobility − which account for categorical data − to understand how the expansion of public health insurance may be related to both health inequality and mobility	Mexico	Coverage
2021	Constante HM, Marinho GL, Bastos JL.	*The door is open, but not everyone may enter: Racial inequities in healthcare access across three brazilian surveys*	This study estimates three health indicators based on three national surveys conducted in 2008, 2013, and 2019 to assess the impact of changes in the National Primary Care Policy on racial health disparities	Brazil	Access
2021	Dantas M, et al.	*Fatores associados ao acesso precário aos serviços de saúde no Brasil*	Analisar os fatores associados ao acesso precário aos serviços de saúde pela população brasileira de 19 anos ou mais	Brazil	Coverage
2021	Özçelik EA, et al.	*A Comparative Case Study: Does the Organization of Primary Health Care in Brazil and Turkey Contribute to Reducing*	This comparative case study analyzes the organization of primary health care (PHC) systems in Brazil and Turkey to draw lessons that can be applied to other countries that may be considering reforming the organization of their	Brazil	Provision

		Disparities in Access to Care?	PHC systems as a way to address health inequalities		
2021	Bautista-González E, et al.	*Health-care guidelines and policies during the COVID-19 pandemic in Mexico: A case of health-inequalities.*	The objective of this study is to conduct an exploratory review of health policies published during the COVID-19 pandemic by health institutions in Mexico	Mexico	Coverage
2021	Gómez F, et al.	*Healthy aging determinants and disability among older adults: SABE Colombia*	To identify the main factors associated with disability among older adults in Colombia, adjusted for the structural and intermediate determinants of healthy aging	Colombia	Coverage
2021	Melo-Ferreira V, Junger WL, Werneck GL.	*Determinantes contextuais e individuais da utilização da triagem auditiva neonatal: Pesquisa Nacional de Saúde, 2013*	O presente estudo avaliou o efeito de determinantes contextuais e individuais da utilização da triagem auditiva neonatal no Distrito Federal e capitais brasileiras	Brazil	Coverage
2021	Oliveira SC, et al.	*Relações público-privadas no sistema de saúde do Chile: regulação, financiamento e provisão de serviços*	Este artigo analisa a configuração das relações público-privadas no sistema de saúde chileno entre 2000 e 2018, com foco em organização e regulação, financiamento e prestação de serviços	Chile	Financing/ Access
2022	Costa DCAR, et al.	*Crise econômica e disparidades no gasto, oferta e utilização de serviços públicos e privados de saúde no Brazil no período entre 2011 e 2019*	Analisa as repercussões da crise econômica sobre os setores público e privado do sistema de saúde brasileiro e realiza uma análise de tendência de indicadores econômicos e assistenciais, elaborados a partir de dados secundários de fontes públicas oficiais, relacionados ao gasto, ao desempenho econômico de planos e seguros de saúde, à oferta e utilização de serviços	Brazil	Financing
2022	Macedo JB, et al.	*Gastos catastróficos em saúde: análise da associação com condições socioeconômicas em Minas Gerais, Brasil*	O objetivo deste estudo foi avaliar os gastos catastróficos em saúde (GCS) e sua associação com condições socioeconômicas nos anos de 2009, 2011 e 2013 em Minas Gerais	Brazil	Financing
2022	Silva B, et al.	*Dual Use of Public and Private Health Care Services in Brazil.*	The purpose of this study is to describe this dual use and assess its relationship with socioeconomic status (SES)	Brazil	Provision
2022	Carvalho-Costa IMN, et al.	*Quality of Life among Patients with Acute Coronary Syndromes Receiving Care from Public and*	The objective of this study was to analyze the quality of life (QOL) of patients with acute coronary syndrome (ACS) who received medical treatment through a public or private healthcare system	Brazil	Coverage

		Private Health Care Systems in Brazil.			
2022	Boer R, et al.	*Acesso e acessibilidade ao rastreamento de câncer em mulheres brasileiras com lesão medular*	Identificar e analisar a acessibilidade e o acesso de mulheres brasileiras com lesão medular para a realização de exames preventivos do câncer de mama e colo de útero	Brazil	Access
2022	Rojas-Roque C, et al.	*Socioeconomic Inequalities in the Prevalence of Diabetes in Argentina: A Repeated Cross-Sectional Study in Urban Women and Men.*	This study measured socioeconomic inequalities in the prevalence of diabetes between 2005 and 2018 in an urban population in Argentina	Argentina	Access
2023	Garcia-Diaz R, et al.	*Equity in out-of-pocket health expenditure: Evidence from a health insurance program reform in Mexico*	Our objective was to analyze the long-term effects of the voluntary health insurance program Seguro Popular (SP) − which was recently discontinued and replaced by the Institute of Health for Well-Being (INSABI) − on GDP equity among the poor in Mexico	Mexico	Financing
2023	Vieira Y, et al.	*Socioeconomic inequities in specialized health services use following COVID-19 in individuals from Southern Brazil.*	The objective of this study was to assess socioeconomic inequalities in the use of specialized health services by health insurance plan and income level following the COVID-19 pandemic among individuals residing in southern Brazil	Brazil	Others
2023	Andrade DAP, et al.	*Discrepancies in breast cancer's oncological outcomes between public and private institutions in the southeast region of Brazil: a retrospective cohort study*	The objective of this study is to assess whether disease-free survival (DFS) and overall survival (OS) differ between public and private specialized centers	Brazil	Coverage
2023	Bourlon MT, et al.	*Impact of healthcare inequities on survival in Mexican patients with metastatic renal cell carcinoma*	The objective of the study was to evaluate the impact of health insurance on access to treatment and survival among patients with metastatic renal cell carcinoma (mRCC)	Mexico	Coverage
2023	Rodríguez Hernández JM, et al.	*Analysis of Inequalities and Inequities in Maternal Mortality in Chocó, Colombia.*	We used a mixed-design study to analyze inequalities and inequities in maternal mortality (MM) in Chocó (Colombia) between 2010 and 2018	Colombia	Coverage
2023	Murillo-Peña JP, et al.	*Medical encounters per capita in the*	The objective of this study is to offer a new perspective on the Peruvian health care system, based	Peru	Provision

		Peruvian health system, 2019. Towards a new description of the Peruvian health system	on the production of public goods in health care		
2023	Gideon J.	*Financing Development Through PPPs: What Does This Mean for Inequalities*	Reflect on the potential challenges and opportunities presented by public-private partnerships (PPPs) in the health sector in Latin America, before focusing on the case of Peru and examining the extent to which PPPs can address the main challenges faced by a health system characterized by fragmentation and inequalities	Latin America	Financing
2023	Ortega F, Pele A.	*Brazil's unified health system: 35 years and future challenges*	2023 marks the 35th anniversary of Brazil's current Constitution (1988), in which health was defined as a universal right and a responsibility of the State. Reflections on this period are presented	Brazil	Provision
2024	Lapo-Talledo GJ.	*Nationwide study of in-hospital maternal mortality in Ecuador, 2015-2022.*	The objective of this study was to analyze estimates of maternal mortality related to hospital deliveries and the sociodemographic factors that influence this mortality in Ecuador between 2015 and 2022	Ecuador	Coverage
2024	Díaz-Ruiz R, et al.	*Socioeconomic inequalities in the use of medical consultation services in Peru, 2019.*	The objective of this study was to analyze and break down socioeconomic inequalities in the use of medical consultation services in Peru	Peru	Coverage
2024	Arias-Ortiz N, et al.	*The Impact of Health Inequities on Population-Based Breast Cancer Survival in a Colombian Population, 2008-2015.*	To obtain breast cancer survival estimates in Manizales, Colombia, taking into account socioeconomic status, health insurance status, and residential area, while adjusting for age, histology, and stage at diagnosis	Colombia	Coverage
2024	Roberti J, et al.	*Inequalities in health system coverage and quality: a cross-sectional survey of four Latin American countries*	We characterized inequalities in the quality of the health care system in Colombia, Mexico, Peru, and Uruguay	Colombia, Mexico, Peru, and Uruguay	Coverage

Source: elaborated by the authors.
